# Supplementary material for: Gestational Hypertension and High-Density Lipoprotein Function: An Explorative Study in Overweight/Obese Women of the DALI Cohort
Source: Antioxidants (Basel). 2022 Dec 29;12(1):68. doi: 10.3390/antiox12010068 (PMC9854490; doi:10.3390/antiox12010068)
Supplement: Supplementary file 1 [file antioxidants-12-00068-s001.zip › antioxidants-2056995-supplementary.pdf]

Supplementary Material:

DALI Study

This is a secondary analysis of the vitamin D and lifestyle intervention for GDM prevention (DALI) multicenter randomized controlled trial study conducted between 2012–2015 in 11 study centers in nine European countries (Austria, Belgium, Denmark (Odense, Copenhagen), Ireland, Italy (Pisa, Padua), Netherlands, Poland, Spain and United Kingdom). The study was registered under trial registration number ISRCTN70595832 and was approved by all local ethics committees.

Pregnant women <20 weeks of gestation with a singleton pregnancy, aged ≥18 years with a pre-pregnancy BMI of ≥29 kg/m2 were invited to participate. Women with GDM at baseline based on International Association of Diabetes and Pregnancy Study Group (IADPSG) criteria (fasting venous plasma glucose ≥5.1 mmol/L and/or 1-hour glucose of ≥10 mmol/L and/or 2-hour glucose of ≥8.5 mmol/L) [29] or who were pre-diagnosed with diabetes, chronic medical conditions, or psychiatric disorders were excluded. Other exclusion criteria were an inability to walk 100 m safely, requirement for a complex diet and not being able to communicate with the lifestyle coach due to a lack of language proficiency.

After written informed consent, women were randomized into either three (pilot study) or four (lifestyle trial) groups receiving counselling for Healthy Eating (HE), Physical Activity (PA), Healthy Eating + Physical Activity (HE+PA) and - in the lifestyle trial - a control group receiving usual care (UC). For this analysis, data from 192 participants were combined into one cohort and analyzed observationally. The participants of whom samples from all three time points in pregnancy (<20 weeks, 24-28, and 35-37 weeks of gestation) were available were used in the analysis.

Data collection

Data were collected from participants at three time points: baseline (<20 weeks), at 24–28 weeks, at 35–37 weeks. Cord blood was collected immediately after birth. Maternal data collection included anthropometrics (weight, height), blood samples and questionnaire data (including age, ethnicity, parity and smoking status). Additional information on pre-pregnancy weight, maternal/paternal smoking, alcohol consumption and medical history was collected by questionnaire.

Sample collection and storage

At baseline, women underwent assessments and fasting blood samples were collected prior to and during an OGTT. This was repeated at 24-28 weeks and at 35-37 weeks. From women with GDM at 24-28 weeks only fasting blood samples were taken at 35-37 weeks, as previously described [25,26]. Whole blood samples were separated into serum and stored at -20 °C or -80 °C to be further handled in the ISO-certified central trial laboratory in Graz, Austria.

Supplementary Table S1. Characteristics of women selected for analyses and those who were excluded.

Maternal characteristics	Selected N=192	Non-selected N=429	P
Age, years n=621	31.7 ± 5.4	31.9 ± 5.4	0.73
Prepregnancy BMI, kg/m^2 n=621	34.1 ± 4.5	33.6 ± 4.1	0.16
Gestational weight gain, kg n=491	8.4 ± 4.8	7.6 ± 4.6	0.05
Primiparous n=621	102 (53%)	216 (50%)	0.42
Married/living with partner n=621	181 (94%)	399 (93%)	0.56
High education n=621	**119 (62%)**	**216 (50%)**	**0.01**
European descent n=621	170 (89%)	120 (88%)	0.87
Smoking n=618	**18 (9%)**	**80 (19%)**	**0.003**
GDM n=499	**55 (29%)**	**119 (39%)**	**0.03**
GHTN n=525	24 (13%)	36 (11%)	0.41
Preeclampsia n=526	6 (3%)	11 (3%)	0.99
Neonatal characteristics			
Birthweight n=554	**3575 ± 520**	**3427 ± 568**	**0.003**
Gestational age at birth n=553	39.7 ± 1.4	39.4 ± 2.9	0.16
Female sex n=560	90 (47%)	179 (49%)	0.69

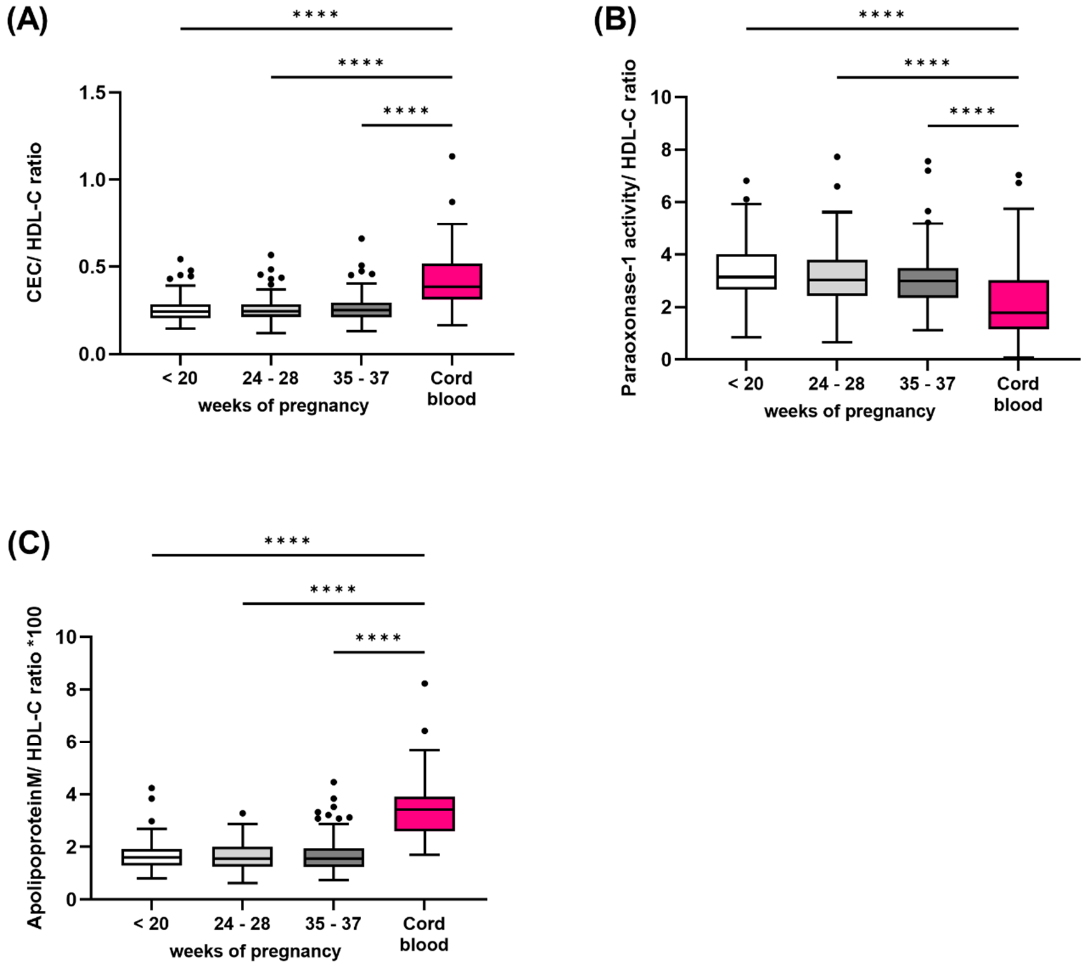

Suppl. Figure S1. HDL-functionalities corrected for HDL-C levels in the DALI cohort. **(A)** Ratio of HDL cholesterol efflux capacity (CEC) and HDL-C. Paraoxonase-1 activity **(B)** and serum levels of apolipoprotein M **(C)**. Data are presented as Tukey-Boxplots, showing the median and interquartile ranges, as well as minimum, maximum values and outliers. ****p<0.0001.

References

25. Jelsma, J.G.M.; van Poppel, M.N.M.; Galjaard, S.; Desoye, G.; Corcoy, R.; Devlieger, R.; van Assche, A.; Timmerman, D.; Jans, G.; Harreiter, J.; et al. DALI: Vitamin D and Lifestyle Intervention for Gestational Diabetes Mellitus (GDM) Prevention: An European Multicentre, Randomised Trial—Study Protocol. *BMC Pregnancy Childbirth* **2013**, *13*, 142. https://doi.org/10.1186/1471-2393-13-142.
26. Simmons, D.; Jelsma, J.G.M.; Galjaard, S.; Devlieger, R.; Van Assche, A.; Jans, G.; Corcoy, R.; Adelantado, J.M.; Dunne, F.; Desoye, G.; et al. Results from a European Multicenter Randomized Trial of Physical Activity and/or Healthy Eating to Reduce the Risk of Gestational Diabetes Mellitus: The DALI Lifestyle Pilot. *Diabetes Care* **2015**, *38*, 1650–1656. https://doi.org/10.2337/dc15-0360.
29. Bosteen, M.H.; Dahlbäck, B.; Nielsen, L.B.; Christoffersen, C. Protein Unfolding Allows Use of Commercial Antibodies in an Apolipoprotein M Sandwich ELISA. *J. Lipid Res.* **2015**, *56*, 754–759. https://doi.org/10.1194/jlr.D055947.
